# Supplementary material for: Future habitat changes of Bactrocera minax Enderlein along the Yangtze River Basin using the optimal MaxEnt model
Source: PeerJ. 2023 Nov 21;11:e16459. doi: 10.7717/peerj.16459 (PMC10668831; doi:10.7717/peerj.16459)
Supplement: Supplemental Information 1 [file peerj-11-16459-s001.docx]

**Table S1. 20 initial environmental variables used in this study**

|  | Variables and description | Unit |
| --- | --- | --- |
| bio1 | Annual Mean Temperature | ℃ |
| bio2 | Mean Diurnal Range(Mean of monthly (max temp - min temp) | ℃ |
| bio3 | Isothermality (BIO2/BIO7) (* 100) | / |
| bio4 | Temperature Seasonality (standard deviation *100) | ℃ |
| bio5 | Max Temperature of Warmest Month | ℃ |
| bio6 | Min Temperature of Coldest Month | ℃ |
| bio7 | Temperature Annual Range (BIO5-BIO6) | ℃ |
| bio8 | Mean Temperature of Wettest Quarter | ℃ |
| bio9 | Mean Temperature of Driest Quarter | ℃ |
| bio10 | Mean Temperature of Warmest Quarter | ℃ |
| bio11 | Mean Temperature of Coldest Quarter | ℃ |
| bio12 | Annual Precipitation | mm |
| bio13 | Precipitation of Wettest Month | mm |
| bio14 | Precipitation of Driest Month | mm |
| bio15 | Precipitation Seasonality (Coefficient of Variation) | / |
| bio16 | Precipitation of Wettest Quarter | mm |
| bio17 | Precipitation of Driest Quarter | mm |
| bio18 | Precipitation of Warmest Quarter | mm |
| bio19 | Precipitation of Coldest Quarter | mm |
| alt | Altitude | m |
